# The impact of community paramedicine service on prehospital time intervals in rural Norway: a quasi-experimental study

**DOI:** 10.1186/s12873-026-01642-4

**Published:** 2026-07-07

**Authors:** Odd Eirik Elden, Oddvar Uleberg, Emma Brulin, Bodil J. Landstad, Maja Pålsdatter Lønvik, Øyvind Salvesen, Havard Dalen

**Affiliations:** 1 Department of Circulation and Medical Imaging, Faculty of Medicine and Health Sciences, Norwegian University of Science and Technology, Trondheim University Hospital, Trondheim, Norway; 2 Department of Prehospital Services, Nord-Trøndelag Hospital Trust, Levanger, N-7600 Norway; 3 Department of Emergency Medicine and Prehospital Services, St Olavs University Hospital, Trondheim, Norway; 4 Department of Surgery, Levanger Hospital, Nord-Trøndelag Hospital Trust, Levanger, Norway; 5 Department of Research and Development, Norwegian Air Ambulance Foundation, Oslo, Norway; 6 Unit of Occupational Medicine, Institute of Environmental Medicine, Karolinska Institutet, Stockholm, Sweden; 7 Centre for Occupational and Environmental Medicine, Region Stockholm, Stockholm, Sweden; 8 Department of Human Sciences, Division of Public Health Sciences, Mid Sweden University, Östersund, Sweden; 9 Department of Public Health and Clinical Medicine, Umeå University, Umeå, Sweden; 10 Unit of Research, Region Jämtland Härjedalen, Östersund, Sweden; 11 Department of Public Health and Nursing, Norwegian University of Science and Technology, Trondheim, Norway; 12 Department of Medicine, Levanger Hospital, Nord-Trøndelag Hospital Trust, Levanger, Norway; 13 Department of Cardiology and Cardiothoracic Surgery, St Olavs University Hospital, Trondheim, Norway

**Keywords:** Prehospital emergency care, Rural health services, Time study, Community paramedicine

## Abstract

**Background:**

Community paramedicine service (CPS) may improve prehospital healthcare delivery in rural regions. As CPS does not provide patient transport, its introduction could prolong prehospital time intervals. Thus, we aimed to evaluate whether CPS implementation influenced prehospital time intervals for acute patients admitted to hospital.

**Methods:**

In this quasi-experimental study, we compared prehospital time intervals for acute missions resulting in hospital admission in two rural Norwegian municipalities with similar characteristics. Data were collected over four years before and two years after CPS implementation in one municipality (CPS_REGION_). The second municipality served as a control (CONTROL_REGION_).

**Results:**

Of 1,872 missions, 362 acute missions with hospital admission were included, of which 228 occurred in the CPS_REGION_ and 134 in the CONTROL_REGION_. After CPS implementation, the median response interval was reduced from 19 to 11 min (*p* < 0.001), whereas no statistically significant changes were observed for the other prehospital time intervals in the CPS_REGION_ or for any prehospital time intervals in the CONTROL_REGION_ (all *p* > 0.12). Difference-in-differences analyses showed no statistically significant difference for total prehospital interval (-2 min, *p* = 0.850), while there were significantly shorter response- (-7 min, *p* < 0.001) and emergency medical service (EMS) on-scene intervals (-6 min, *p* = 0.003). Higher age was associated with longer total prehospital interval (*p* = 0.018).

**Conclusions:**

CPS implementation was not associated with changes of the total prehospital interval but was linked to shorter response interval and EMS on-scene intervals, which may indicate earlier arrival of prehospital personnel without evidence of delayed start of transport. However, given the observational design and the absence of clinical outcome data, the overall impact on patient safety and system performance remains uncertain.

## Introduction

Community paramedicine service (CPS), in which advanced paramedics provide community-based healthcare using ambulance-type vehicles without stretchers, represents an innovative and resource-efficient supplement to standard emergency medical services (EMS). CPS has been shown to reduce emergency department (ED) visits [1], thereby alleviating pressure on prehospital services driven by ageing populations, urbanization, and constrained healthcare funding [2–4]. CPS is typically staffed by a single EMS professional with additional training [5]. Various CPS models have been successfully implemented in both rural and urban settings in countries such as Australia, Canada, the United Kingdom, and the USA [5–9]. Community paramedicine should be distinguished from related concepts such as non-conveyance models (i.e. patients not transported to hospital by regular EMS) and extended paramedic roles (i.e. personnel with expanded tasks beyond traditional emergency- and transport medicine), although these are often overlapping in practice [1, 9, 10].

Wilkinson-Stokes et al. reported reductions in patient transport to the ED ranging from 14% to 78% following CPS implementation [10]. These benefits extend beyond cost savings and include reduced ED crowding, fewer hospital-associated complications (e.g. discomfort, delirium, infections, and missed medications), and increased EMS availability for time-critical emergencies [10]. Prehospital time intervals – particularly response intervals – are commonly used quality indicators in time-critical emergencies such as cardiac arrest, respiratory failure, trauma, acute coronary syndrome, and stroke [11–16]. Although shorter response intervals are associated with improved outcomes in selected conditions, most emergency missions are not highly time-sensitive [12–15]. Despite the advantages of fewer ED admissions and reduced need for patient transports with CPS, it is essential to ensure that CPS does not prolong prehospital time intervals for patients requiring hospital admission, as this may compromise patient safety. As CPS’ vehicles lack the capability for patient transport, special concerns relate to whether CPS may negatively impact EMS on-scene intervals or delay the start of transport towards hospital.

In 2021, CPS was introduced in a rural municipality in Central Norway (CPS_REGION_). This study aimed to evaluate whether CPS implementation affected prehospital time intervals (response interval, EMS on-scene interval and total prehospital interval) using data collected before and after the implementation, compared with a similar municipality without CPS (CONTROL_REGION_). As the CPS is located closer to the patients and can initiate assessment and treatment on scene despite lacking transport capability, we hypothesised that CPS introduction would reduce response intervals without prolonging other prehospital time intervals for acute patients admitted to hospital.

## Methods

### Study design

We conducted a quasi-experimental pre- and post-intervention study comparing prehospital time intervals in two rural Norwegian municipalities over six years: four years before and two years after the implementation of CPS in one municipality (CPS_REGION_). The second municipality served as a control (CONTROL_REGION_) and maintained standard EMS throughout the study period. No randomization was performed, as the regions represented naturally occurring settings. Reporting followed the STROBE checklist for cohort studies [17].

### Study setting

Both municipalities were rural, defined by the Norwegian Directorate of Health as having fewer than 10,000 inhabitants [18]. They were characterised by agricultural lowlands surrounded by fjords, rivers and mountains, with sparse populations and small towns. As shown previously, CPS_REGION_ and CONTROL_REGION_ were comparable with respect to socioeconomic and health-related characteristics [19]. Descriptions of the two municipalities are presented in Table 1.

**Table 1:** Descriptions of the two municipalities

	CPS_*REGION*_ (Inderøy)	CONTROL_*REGION*_ (Overhalla)	Norway
Inhabitants per 01.04.2022, *n*	6,832	3,889	5,514,042
Mean age, per 01.01.2022, *years*	42.9	40.7	41.0
Distances, *km*			
-ambulance station to health centre*	23	23	
-average distance to hospital^#^	35	19	
Geographical area size, *in km*^*2*^	366	730	385,207

*Health centre with a general practitioner office, physiotherapist and nurses. ^#^Estimated from aggregated data. Abbreviations: CPS_REGION_, municipality implementing CPS between periods; CONTROL_REGION_, control municipality; km, kilometre; n, number

None of the municipalities had local ambulance stations; EMS was dispatched from neighbouring municipalities. All EMS and CPS missions were coordinated by the Emergency Medical Communication Centre (EMCC), Nord-Trøndelag Hospital Trust, using the Norwegian Index for Medical Emergency Assistance to determine urgency levels (acute, urgent, non-urgent) [20]. Acute responses indicate life-threatening situations, urgent responses involve potential deterioration, and non-urgent responses involve planned or non-time-critical missions [20]. According to national regulations, acute EMS responses in rural areas are expected to reach the scene within 25 min [20].

### Community paramedicine service (CPS)

**Fig. 1:**
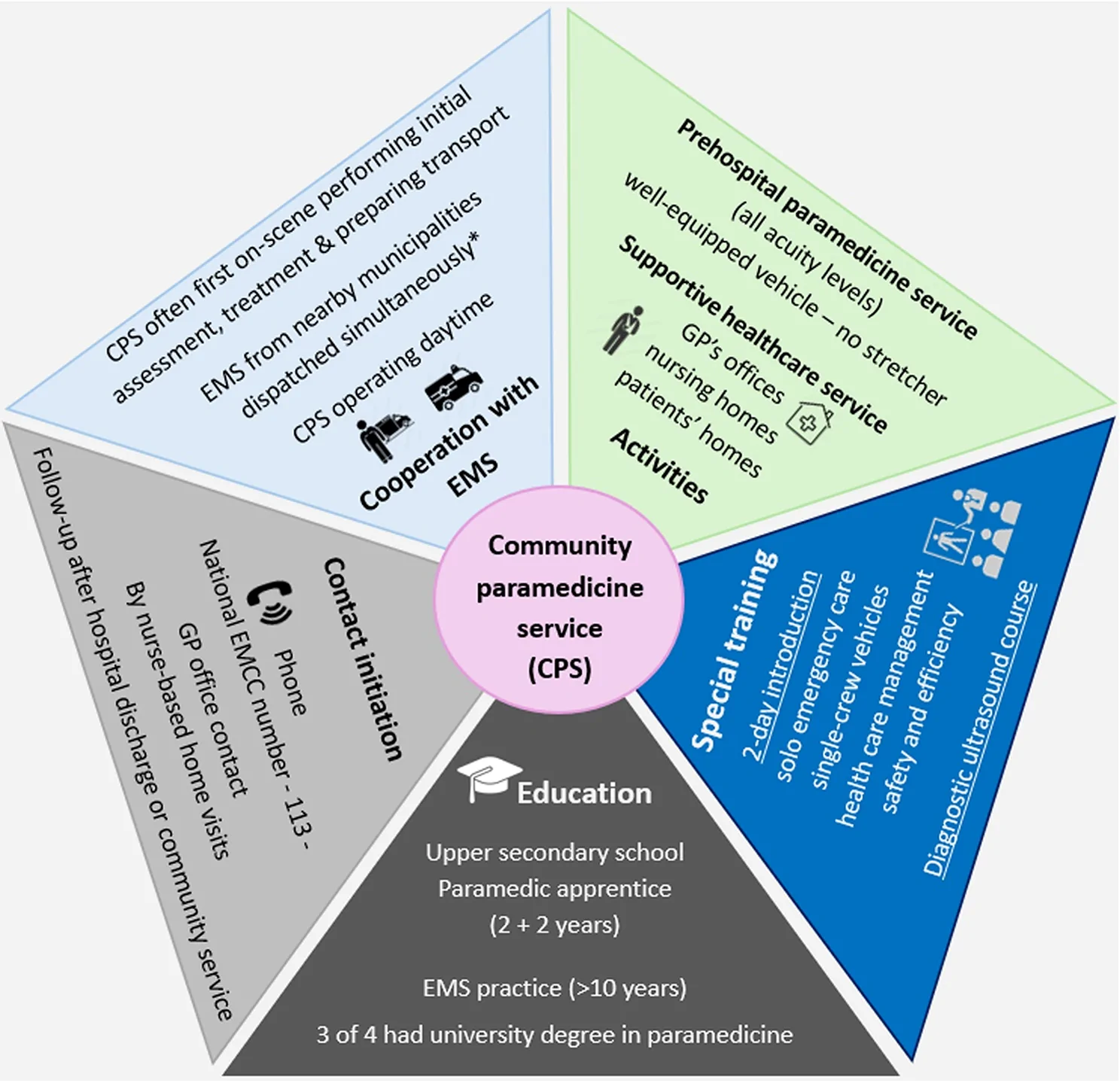
Community paramedicine service system overview. Overview of five central pillars for the community paramedicine service studied; with adequately educated and trained personnel, contact information and dispatch system, cooperation with EMS from nearby municipalities and description of activities performed. *EMS and CPS dispatched simultaneously for acute missions in the CPS_REGION_ during period 2. Abbreviations: CPS, community paramedicine service; CPS_REGION_, the municipality implementing CPS between period 1 and 2; EMCC, Emergency Medical Communication Centre; EMS, Emergency Medical Services; GP: general practitioner

The CPS was introduced in the CPS_REGION_ on 1 November 2021 and operated on weekdays from 08:00 to 16:00 as a 2.5-year pilot project. The service was staffed by a paramedic operating an ambulance-type vehicle without a stretcher. Each paramedic held a shared position, working 50% with CPS and 50% with EMS within Nord-Trøndelag Hospital Trust (Fig. 1).

For acute missions in the CPS_REGION_ during weekday daytime hours (08:00–16:00) in period 2, the EMCC dispatched both CPS and EMS simultaneously, while for missions classified as urgent or non-urgent missions, just the CPS was dispatched. The CONTROL_REGION_ relied exclusively on standard EMS 24/7 throughout the study period.

### Data collection

**Fig. 2:**
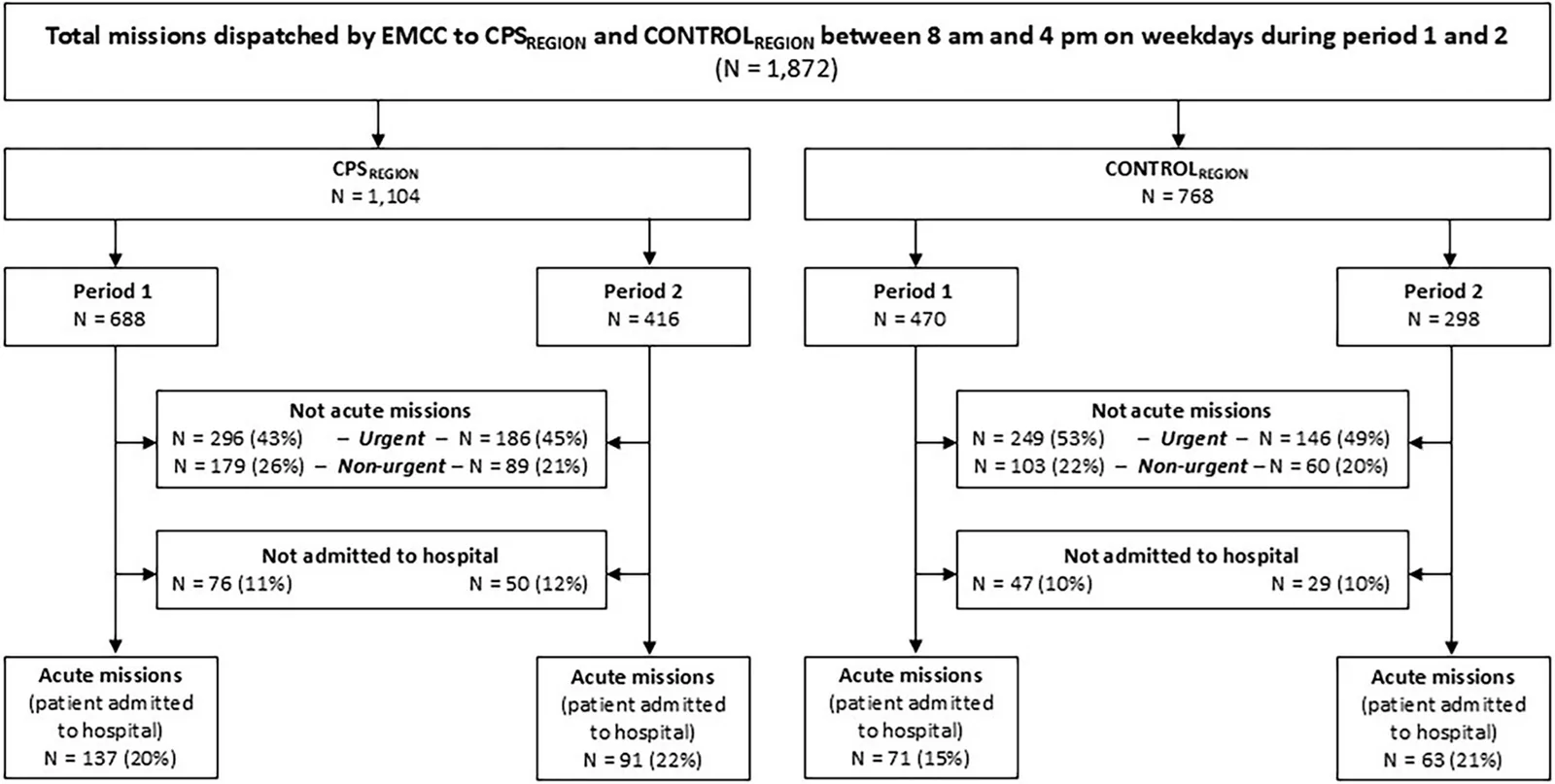
Flowchart for included acute level missions leading to hospital admission. Abbreviations: CONTROL_REGION_, control municipality not implementing CPS; CPS_REGION_, municipality implementing CPS between period 1 and 2; EMCC, Emergency Medical Communication Centre; N, number

Prehospital time intervals for EMS and CPS missions were extracted from EMCC records using the national Acute Medical Information System (AMIS; CSAM Health AS, Oslo, Norway) [20]. Data included patient identifiers, location, urgency level, response interval, on-scene interval, and total prehospital interval. Time points were digitally recorded at dispatch, on-scene arrival, and departure, a method shown to be more accurate than manual reporting [21]. Only acute missions during weekdays (08:00–16:00) resulting in hospital admission were included. Missions resulting in non-hospital transport or cases in which the patient was pronounced dead on scene were excluded (Fig. 2).

Data were collected for the pre-intervention period (1 January 2016 to 31 December 2019) and the post-intervention period (1 November 2021 to 31 October 2023). The intervening period was excluded to avoid confounding related to the COVID-19 pandemic. Period 1 was prior to the COVID-19 pandemic, and period 2 was after most of the restrictions on public life and healthcare providers were lifted. However, the rate of hospital admissions related to COVID-19 does not explain the increase in activity seen in both CPS_REGION_ and CONTROL_REGION_ during the last study period.

### Outcome measurements

The following prehospital time intervals served as outcome measures: First, *the response interval* was measured as the time from EMCC call to CPS or EMS arrival on scene. Second, the *EMS on-scene interval* was measured from EMS arrival on scene to the start of transport towards the hospital. Third, the *total prehospital interval* was measured from the EMCC call to the start of transport towards the hospital by EMS. The start of transport was chosen as the end of the total prehospital interval, as the average transport distance to the hospital differed significantly between municipalities. All time intervals were recorded in minutes.

### Statistical analysis

The prehospital time intervals were not normally distributed, and thus, they were presented as median (interquartile ranges shown by the 25th − 75th percentiles (IQR)). Proportions were presented as numbers and percentages. Non-parametric Mann–Whitney U tests were used to compare within-regions time intervals across periods in unadjusted analyses using IBM SPSS Statistics (version 30; IBM Corp., Armonk, NY, USA).

A difference-in-differences analytical approach was used to evaluate the impact of CPS implementation on prehospital time intervals by comparing temporal changes between the intervention and control groups. Pre-intervention trends were explored by examining annual data across the four years of period 1 using the Jonckheere-Terpstra test, as a limited assessment of the parallel trends assumption. Prehospital time outcomes were analysed using generalised linear models (GLMs). Among the candidate models, a negative binomial regression model was selected because it demonstrated superior model fit and appropriately accounted for overdispersion, with variance exceeding the mean. We compared the pre/post time intervals in the CPS_REGION_ against the changes in the CONTROL_REGION_ by fitting negative binomial GLMs with identity link using the MASS R package (R Foundation, Salt Lake City, UT, USA). The variables period (pre/post time), region, the interaction between period and region, age and sex were included in the models. Adjustment was limited to these variables since factors as case severity, operational workload, and dispatch prioritisation were not available. Model estimates were tabulated as estimated mean ± standard error in minutes. Difference-in-differences estimates with 95% confidence intervals (95% CIs) were provided by the fully adjusted negative binomial model. The interaction between region and period represents the difference-in-differences estimator and was used to evaluate the outcomes. For all analyses, a p-value < 0.05 was interpreted as statistically significant.

## Results

### Study population

362 acute missions resulting in hospital admission were included: 228 in the CPS_REGION_ and 134 in the CONTROL_REGION_ (Fig. 2). The proportion of acute hospital admissions per inhabitant was similar between regions (3.3% vs. 3.4%). Demographic characteristics are summarized in Table 2.

**Table 2:** Demographic and descriptive data of acute patients transported to the hospital

	CPS_*REGION*_ (period 1)	CPS_*REGION*_ (period 2)	CONTROL_*REGION*_ (period 1)	CONTROL_*REGION*_ (period 2)
Patients, *n*	137	91	71	63
Female sex, n (%)	68 (49.6)	38 (41.8)	34 (47.9)	28 (44.4)
Age, median (IQR), years	68 (48–83)	71 (55–81)	65 (44–74)	76 (63–82)
Patient categories				
• Disease, n (%)	111 (81.0)	76 (83.5)	59 (83.1)	52 (82.6)
• Injury, n (%)	22 (16.1)	13 (14.3)	11 (15.5)	11 (17.4)
• Intoxication, n (%)	4 (2.9)	2 (2.2)	1 (1.4)	-

Data for both periods are limited to weekdays between 08:00 and 16:00; period 1 lasted 4 years, while period 2 lasted 2 years. Abbreviations: CPS_REGION_, municipality implementing CPS between periods; CONTROL_REGION_, control municipality; IQR, interquartile range; n, number

### CPS and prehospital time intervals

In Table 3, median (IQR) prehospital time intervals are shown. In unadjusted analyses, a reduction in response interval was observed from period 1 to period 2 in the CPS_REGION_ (*p* < 0.001), while no statistically significant changes were observed for EMS on-scene interval or total prehospital interval (both *p* ≥ 0.34). Similarly, no statistically significant changes were observed in the CONTROL_REGION_ (all *p* > 0.12).

**Table 3:** Prehospital time intervals for acute patients transported to hospital

	Response interval		EMS on-scene interval		Total prehospital interval	
CPS_*REGION*_ (period 1)	19 (16–26)	*p* < 0.001	14 (7–24)	*p* = 0.34	35 (26–45)	*p* = 0.54
CPS_*REGION*_ (period 2)	11 (5–17)		12 (7–18)		35 (30–45)	
CONTROL_*REGION*_ (period 1)	20 (17–24)	*p* = 0.12	18 (13–25)	*p* = 0.57	40 (32–47)	*p* = 0.68
CONTROL_*REGION*_ (period 2)	19 (16–22)		20 (10–30)		38 (32–48)	

Data (minutes) are presented as median (interquartile range). P-values annotate differences across periods within regions. Definitions: Response interval, time from EMCC call to CPS or EMS arrival on scene; EMS on-scene interval, time from EMS arrival on scene to the start of transport towards hospital; total prehospital interval, time from EMCC call to start of transport towards hospital. Abbreviations: CPS_REGION_, municipality implementing CPS between periods; CONTROL_REGION_, control municipality

During the four years prior to CPS implementation the annual median (IQR) for all prehospital intervals remained relatively stable in both regions with no linear trends (Table 4).

**Table 4:** Prehospital time interval trends in CPS_REGION_ and CONTROL_REGION_ in period 1

Year	Response interval		EMS on-scene interval		Total prehospital interval	
	CPS_REGION_	CONTROL _REGION_	CPS_REGION_	CONTROL _REGION_	CPS_REGION_	CONTROL _REGION_
2016	19 (17–28)	21 (19–24)	12 (6–28)	16 (12–24)	38 (26–49)	38 (34–44)
2017	18 (16–26)	19 (15–23)	18 (8–27)	19 (14–29)	43 (28–46)	42 (32–49)
2018	20 (13–27)	20 (16–23)	12 (7–24)	20 (13–27)	38 (25–45)	40 (32–49)
2019	20 (17–24)	22 (17–24)	14 (7–20)	18 (13–21)	32 (26–43)	41 (32–43)
*p for trend*	*0.964*	*0.567*	*0.502*	*0.673*	*0.267*	*0.853*

Intervals in minutes are presented as median (IQR). P-values shown for trends according to the regions for each of the prehospital time intervals. Abbreviations: CPS_REGION_, municipality implementing CPS between periods; CONTROL_REGION_, control municipality

During period 2 in the CPS_REGION_, CPS had a shorter median response interval than EMS (11 vs. 23 min) and was first on scene in 82 of 91 acute missions (90.1%). On average the CPS arrived at the scene 11 min (SD 4) before the EMS.

**Fig. 3:**
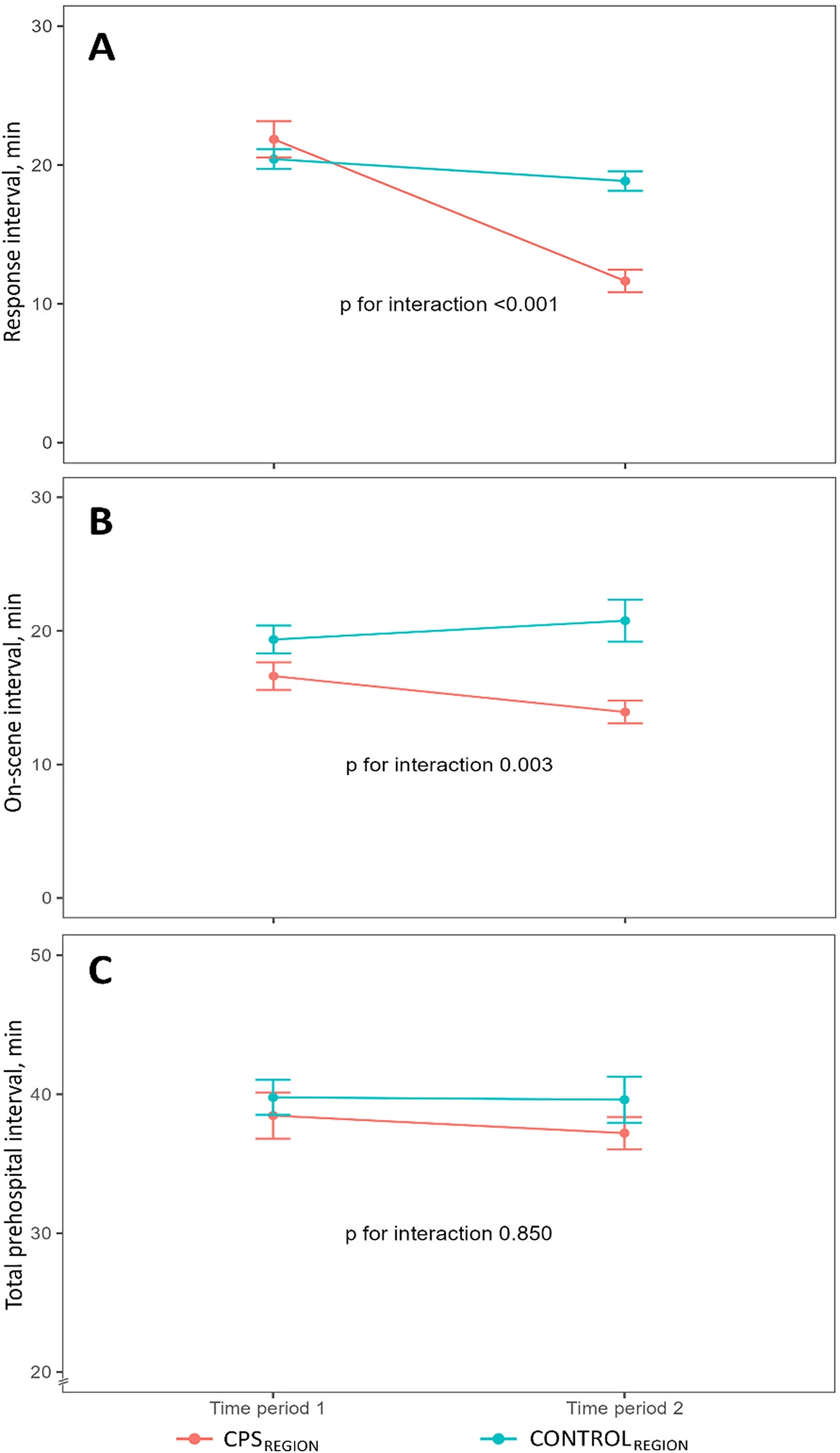
Difference-in-differences plots of adjusted prehospital time intervals. Data represent adjusted mean values and standard error bars from general linear models adjusted for age and sex. (**A**) Response interval, (**B**) On-scene interval, and (**C**) Total prehospital interval. Divergence between the CPS_REGION_ and CONTROL_REGION_ from period 1 to period 2 represents the difference-in-differences estimate and is indicated by the p-value for the interaction. Abbreviations: CONTROL_REGION_, control municipality not implementing CPS; CPS_REGION_, municipality implementing CPS between period 1 and 2; min, minutes

The difference-in-differences estimates are shown in Table 5. In analyses adjusted for sex and age, no statistically significant difference-in-differences estimate was observed for total prehospital interval (-2 min, *p* = 0.850), response interval was reduced by 7 min (*p* < 0.001) and EMS on-scene interval was reduced by 6 min (*p* = 0.003) aligned with CPS implementation (Fig. 3). Higher age was associated with a longer total prehospital interval (*p* = 0.018) but not with the other prehospital time intervals. Sex was not significantly associated with any prehospital time interval.

**Table 5:** Difference-in-differences estimates for prehospital time intervals

	Response interval (minutes)	EMS on-scene interval (minutes)	Total prehospital interval (minutes)
CPS_REGION_, period 1	21.84 ± 1.80	16.61 ± 1.74	38.46 ± 1.66
CPS_REGION_, period 2	11.64 ± 1.28	13.92 ± 1.82	39.77 ± 1.26
CONTROL_REGION_, period 1	20.42 ± 2.35	19.35 ± 2.77	37.20 ± 1.16
CONTROL_REGION_, period 2	18.84 ± 2.32	20.76 ± 3.14	39.60 ± 1.66
DiD estimates, adjusted	-7.14 (-9.77, -4.51)	-6.44 (-10.10, -2.84)	-1.84 (-6.28, 2.60)
P-values, interaction	< 0.001	0.003	0.850
P-values, age	0.182	0.554	0.018
P-values, sex	0.124	0.319	0.067

Difference-in-differences estimates obtained using generalised linear models with negative binomial distribution and identity link adjusted for age and sex. Descriptive data shown as unadjusted estimated means ± standard errors. Abbreviations: CPS_REGION_, municipality implementing CPS between period 1 and 2; CONTROL_REGION_, control municipality not implementing CPS between period 1 and 2; DiD, Difference-in-differences assessed as the interaction between period and region

## Discussion

This quasi-experimental study evaluated the impact of CPS on rural prehospital time intervals. Implementation of a CPS was not associated with a change in the total prehospital interval but was associated with significant reductions in both response interval and EMS on-scene interval. These findings may indicate earlier access to prehospital assessment and care without evidence of delayed initiation of patient transport. Overall, the results indicate that CPS primarily influences early stages of the prehospital care pathway, while later stages appear constrained by factors not directly modified by the intervention, such as geography and transport logistics [11, 16].

### Interpretation of findings

The observed reduction in response interval is likely explained by the geographical positioning and daytime availability of CPS personnel. Decentralised deployment of prehospital resources has previously been associated with shorter response times in rural systems [14, 20]. Despite simultaneous dispatch of CPS and EMS, CPS arrived first in 90% of cases, suggesting that local availability can reduce time to initial patient contact. Earlier arrival may facilitate more timely assessment and initiation of care, which is particularly important in rural areas where transport distances are longer [16].

The reduction in EMS on-scene time may reflect improved efficiency following CPS involvement. One plausible explanation is that CPS initiated early assessment and preparatory tasks prior to EMS arrival, facilitating faster handover and decision-making. Similar mechanisms have been suggested in studies of expanded paramedic roles and integrated prehospital models of care [1, 5, 9]. However, this interpretation remains speculative, as detailed process-level data were not available. Alternative explanations, including differences in case mix, operational workload, or dispatch prioritisation, cannot be excluded [14]. Furthermore, the observational design precludes causal inference, and the assumption of parallel pre-intervention trends between regions cannot be formally tested.

Despite improvements in these early phases, total prehospital time remained unchanged. This may reflect a redistribution of time within the prehospital trajectory, where gains in early access are offset by structural constraints later in the pathway, such as transport distances and system capacity. Previous research has shown that time components in prehospital care are interdependent, and improvements in one phase do not necessarily translate into reductions in overall time [11, 22].

### Alignment with literature

Our findings are consistent with previous studies indicating that CPS can be integrated into EMS systems without delaying the initiation of patient transport [19, 23–25]. Much of the existing literature on CPS has focused on reductions in emergency department utilisation and conveyance rates [10], with relatively limited attention to prehospital time intervals. The present study extends this evidence by demonstrating that CPS may improve responsiveness without adversely affecting downstream processes. This aligns with research suggesting that while response time is an important performance indicator, its clinical relevance is primarily limited to time-critical conditions such as cardiac arrest [11–13, 26]. For many emergency presentations, timely assessment and initiation of appropriate care may be at least as important as rapid transport to hospital [22].

At the same time, the lack of reduction in total prehospital time highlights that improvements in individual process measures do not necessarily lead to gains in overall system efficiency. Similar patterns have been described in studies evaluating EMS performance, where focusing on single metrics such as response time can overlook broader aspects of care quality and system functioning [11, 22, 27].

### Implications

The findings may indicate that CPS allowed for earlier access to prehospital assessment and care in rural areas without introducing delays in transport initiation. This supports the role of CPS as a complementary component within existing EMS systems, particularly in settings facing increasing demand due to ageing populations and rising multimorbidity [2–4, 28, 29]. In the present study, median patient age increased between periods and was associated with higher admission rates and longer total prehospital intervals. Combined with a higher volume of missions, this may contribute to time delays due to increased workload. Timely hospital treatment remains crucial for critically ill patients with time-sensitive conditions [16].

However, time-based performance measures represent only one dimension of healthcare quality [27]. Future studies should incorporate patient-centred and safety-related outcomes, including morbidity, mortality, re-contacts, and patient-reported outcomes, to provide a more comprehensive assessment of CPS impact. In addition, further research is needed to better understand the mechanisms through which CPS affects prehospital workflow, including team coordination, clinical decision-making, and resource utilisation [5, 30].

### Limitations

This study has several limitations. First, the observational design precludes causal inference, and residual confounding cannot be excluded. Although the difference-in-differences approach may account for time-invariant differences, adjustment was limited to age and sex, and unmeasured factors such as case severity, underlying clinical conditions, operational workload, dispatch prioritisation, logistics, and changes in case mix may have influenced the results. Second, the study was restricted to acute missions during weekday daytime hours resulting in hospital admission. This restriction reflected the operational hours of CPS and improved comparability between groups but might have introduce selection bias and limits generalisability to other mission types, non-admitted patients, evenings, nights, and weekends. Third, differences in transport distances required defining the total prehospital interval as time to transport initiation rather than hospital arrival, which may affect comparability with other studies.

Fourth, CPS was implemented as a 2.5-year pilot project, limiting the post-intervention period to two full years. Implementation fidelity, adaptation over time, concurrent system-level changes, or secular trends unrelated to CPS may have influenced the observed effects. Finally, the absence of clinical outcome data limits conclusions regarding patient safety and effectiveness.

## Conclusions

CPS implementation in a rural setting was associated with shorter response interval and EMS on-scene intervals but not with changes in total prehospital time. These findings may indicate that implementing CPS provided earlier access to prehospital assessment and care without delaying initiation of patient transport. However, given the observational design and lack of patient-centred outcomes, the impact on clinical effectiveness and system performance remains uncertain.

## Data Availability

The entire deidentified dataset; data dictionary and analytic code for this investigation are available upon reasonable request, from the date of article publication by contacting Odd Eirik Elden, MD, at email [oddeirik.elden@helse-nordtrondelag.no].

## Abbreviations

CI: Confidence interval
CONTROL_*REGION*_: Municipality without change of prehospital emergency medical service
CPS: Community Paramedicine Service
CPS_*REGION*_: Municipality where CPS was introduced between the two periods
DiD: Difference-in-differences
ED: Emergency Department
EMCC: Emergency Medical Communication Centre
EMS: Emergency Medical Service
GP: General Practitioner
IQR: Interquartile range
